# Histone Deacetylase Inhibition in Non-small Cell Lung Cancer: Hype or Hope?

**DOI:** 10.3389/fcell.2020.582370

**Published:** 2020-10-09

**Authors:** Hirva Mamdani, Shadia I. Jalal

**Affiliations:** ^1^Department of Oncology, Karmanos Cancer Institute, Detroit, MI, United States; ^2^Department of Internal Medicine, Division of Hematology/Oncology, Indiana University, Indianapolis, IN, United States

**Keywords:** histone deacetylase inhibitors, NSCLC, HDAC, vorinostat, epigenetic therapy, entinostat, panobinostat

## Abstract

Epigenetic modulation, including acetylation, methylation, phosphorylation, and ubiquitination, plays a pivotal role in regulation of gene expression. Histone acetylation—a balance between the activities of histone acetyltransferases (HATs) and histone deacetylases (HDACs)—is one of the key epigenetic events. Our understanding of the role of HDACs in cancer is evolving. A number of HDAC isoenzymes are overexpressed in a variety of malignancies. Aberrant histone acetylation is associated with dysregulation of tumor suppressor genes leading to development of several solid tumors and hematologic malignancies. Pre-clinical studies have demonstrated that HDAC-1 gene expression is associated with lung cancer progression. Histone hypoacetylation is associated with more aggressive phenotype in adenocarcinoma of the lung. HDAC inhibitors (HDACi) have pleiotropic cellular effects and induce the expression of pro-apoptotic genes/proteins, cause cellular differentiation and/or cell cycle arrest, inhibit angiogenesis, and inhibit transition to a mesenchymal phenotype. Consequently, treatment with HDACi has shown anti-proliferative activity in non-small cell lung cancer (NSCLC) cell lines. Despite promising results in pre-clinical studies, HDACi have shown only modest single agent activity in lung cancer clinical trials. HDAC activation has been implicated as one of the mechanisms causing resistance to chemotherapy, molecularly targeted therapy, and immune checkpoint inhibition. Therefore, there is a growing interest in combining HDACi with these agents to enhance their efficacy or reverse resistance. In this paper, we review the available preclinical and clinical evidence for the use of HDACi in NSCLC. We also review the challenges precluding widespread clinical utility of HDACi as a cancer therapy and future directions.

## Introduction

Regulation of gene expression is a finely balanced process essential for maintenance of homeostasis. Epigenetic modulation plays a critical role in this process. In eukaryotic cells, histones comprise the protein backbone for the chromatin and provide a scaffold for various enzymes to regulate the access of RNA polymerase and other transcription factors to their target genes (Glozak and Seto, 2007; Damaskos et al., 2018). Histone acetylation—a balance between the activities of histone acetyltransferases (HATs) and histone deacetylases (HDACs)—is one of the most extensively studied post-translational modifications of histones (Li and Zhu, 2014; Suraweera et al., 2018). HDACs remove the acetyl groups from histones, allowing compacted chromatin to reform and decrease gene transcription (Glozak and Seto, 2007). So far 18 HDACs have been identified in humans and classified into 4 groups (Class I, II, III, and IV) based on their resemblance with yeast HDACs (Suraweera et al., 2018).

**FIGURE 1:**
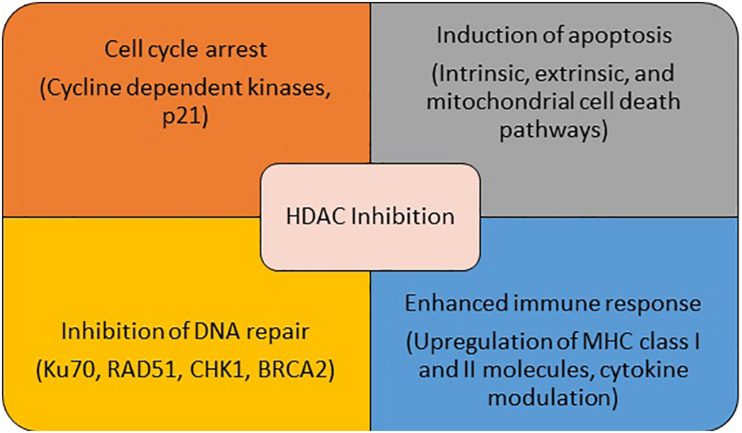
Mechanism of anti-cancer activity of HDAC inhibitors.

Conventional hallmarks of cancer include self-sufficiency in growth signals, evasion of apoptosis, sustained angiogenesis, tissue invasion, and metastasis (Hanahan and Weinberg, 2011). A number of these abnormalities are driven by epigenetic modulation and result from altered activity of one of the key enzymes involved in these processes including HDACs. Several malignant tumors have been shown to have high levels of HDACs (Li and Seto, 2016). Additionally, high expression of various HADCs has been shown to be associated with poor outcomes in patients with a variety of malignancies (Weichert et al., 2008a,b; Oehme et al., 2009; Mithraprabhu et al., 2014). These pre-clinical findings make HDAC a potential target for the treatment of cancer. In addition to its anti-cancer effect via transcription-dependent mechanisms, HDAC inhibition impacts cell proliferation, survival, and angiogenesis via modulation of molecular chaperones, signal transduction proteins, cytoskeletal proteins, cytoplasmic-nuclear transport, and inhibition of hypoxia inducible factors and vascular endothelial growth factor (Glozak et al., 2005; Liang et al., 2006; Witta, 2012). HDAC inhibitors (HDACi) strengthen the immune system by up-regulating the expression of MHC class I and II proteins, and co-stimulatory/adhesion molecules such as CD80, CD86, human leukocyte antigen (HLA)-DR, HLA-ABC, and intracellular adhesion molecule-1 (ICAM-1,28). HDACi may also enhance immune responses by altering the activities of immune cells, either directly or indirectly through cytokine secretion modulation (Miyanaga et al., 2008). The effect of HDACs on tumor metastasis is complex. While HDAC inhibition reversed epithelial-mesenchymal transition via upregulation of E-cadherin, thereby suppressing the tumor’s metastatic potential in some studies, another study showed that inhibition of HDAC11 in breast cancer animal models led to increased migration and egress of tumor cells from lymph nodes to distant sites, via increase in *RRM2* (Witta, 2012; Leslie et al., 2019). Finally, HDACs closely interact with a number of other pivotal cellular pathways and proteins such as DNA repair pathways and heat shock proteins, leading to alteration of a multitude of essential cellular functions by HDACi (Bali et al., 2005; Kiweler et al., 2020). The multiplicity of functions of HDAC suggests potential synergistic role of HDACi with a wide variety of agents used for the treatment of non-small cell lung cancer (NSCLC) (Figure 1). Since certain HDACs are pathologically overexpressed only in tumor cells, HDACi (especially selective HDACi) can be expected to have a reasonable therapeutic window where anti-tumor effect can be obtained with acceptable side effect profile.

Four HDACi are currently approved by the US FDA for the treatment of hematologic malignancies. These include vorinostat and romidepsin for the treatment of cutaneous T cell lymphoma, belinostat for the treatment of peripheral T cell lymphoma, and panobinostat for the treatment of multiple myeloma (Mann et al., 2007; Grant et al., 2010; Sawas et al., 2015; Moore, 2016). Supplementary Table 1 summarizes HDAC class targets of clinically relevant HDACi.

## Altered Histone Modification in NSCLC

Accumulating evidence demonstrates a pivotal role of histone modification in lung carcinogenesis. Lung cancer cells harbor an abnormal pattern of histone modification in comparison with normal lung cells, including hyperacetylation of H4K5/H4K8, hypoacetylation of H4K12/H4K16, and loss of H4K20 trimethylation (Van Den Broeck et al., 2008). Cigarette smoke exposure also influences histone modifications. Nickel, chromate, and arsenite present in tobacco smoke induce H3K4 methylation, which in turn affects the expression of tumor suppressor genes and leads to malignant transformation of the cells (Zhou et al., 2009). Moreover, the majority of squamous cell NSCLC tumors demonstrate elevated levels of HDAC3 (Bartling et al., 2005). Similarly, higher expression of HDAC1 and HDAC3 are associated with poor prognosis in lung adenocarcinoma, while reduced expression of class II HDAC (specifically HDAC5, 6, and 10) is associated with poor prognosis in NSCLC (Osada et al., 2004; Minamiya et al., 2010, 2011). A subset of NSCLC tumor overexpresses FLIP, which blocks the extrinsic apoptotic pathway by inhibiting caspase-8 activation. High cytoplasmic expression of FLIP significantly correlates with shorter overall survival. Treatment with HDACi targeting HDAC1-3 downregulates FLIP expression predominantly via post-transcriptional mechanisms, and results in death receptor- and caspase-8-dependent apoptosis in NSCLC cells, but not in normal lung cells (Riley et al., 2013).

## Role of HDACi in NSCLC—Preclinical Evidence

HDAC inhibition with trichostatin A (TSA) and vorinostat exert strong anti-tumor activity in NSCLC cell lines (Miyanaga et al., 2008). Treatment with TSA leads to activation of intrinsic mitochondrial and extrinsic/Fas/FasL system death pathways and results in dose-dependent apoptosis in H157 lung cancer cells (Kim et al., 2006). Vorinostat leads to upregulation of cyclin-dependent kinase inhibitor p21 in NCI-H520 and NCI-H460 NSCLC cells, G0-G1 cell cycle arrest, and decrease in C-myc and bcl-2 expression (Li et al., 2011). Another HDACi CG200745 has been shown to increased global level of histone acetylation and inhibition of proliferation of NSCLC cells through epigenetic modification of critical genes in cancer cell survival (Chun et al., 2015). Additionally, HDAC6 supports Notch1 signaling in NSCLC cell lines and promotes cell survival and proliferation. Consequently, HDAC6 inhibition leads to G2 arrest, increased apoptosis, and growth inhibition of NSCLC cells (Deskin et al., 2020). Novel HDAC inhibitors, including SL142, SL325, HTPB, and CG0006, demonstrate greater degree of apoptosis of NSCLC cells through induction of caspase-3 activity, disruption of F-actin dynamics, inhibition of mitochondrial membrane potential 2 (MMP2) and MMP9, and increased p21 and p27 expression (Hwang et al., 2009; Han et al., 2010; Shieh et al., 2012). Finally, HDAC inhibition results in downregulation of TNF-alpha receptor-1 mRNA and surface protein expression, leading to attenuated NF-kappa B nuclear translocation. Therefore, HDAC inhibition might exert its therapeutic role by reducing the responsiveness of tumor cells to TNF-alpha mediated activation of NF-kappa B pathway (Imre et al., 2006). This is of particular importance in tumors associated with inflammatory microenvironment, which is the case in many smoking associated NSCLC tumors.

## Clinical Utility of HDACi in NSCLC

Over the past two decades, the therapeutic landscape of NSCLC has evolved significantly to include multiple molecularly targeted therapies and immune checkpoint inhibitors. However, there remains a subset of patients who do not benefit from these therapies. Moreover, the majority of patients eventually experience disease progression following initial response to these therapies. Therefore, there is an urgent need for novel treatment strategies for the treatment of NSCLC. Epigenetic modulation, including HDAC inhibition, is a prospective therapeutic approach, which may evade the challenges of tumor heterogeneity and dependability on targetable molecular alterations.

### HDACi Monotherapy in NSCLC

Despite the plethora of pre-clinical evidence supporting the activity of HDACi in NSCLC, these agents have demonstrated only modest single agent efficacy in clinical trials. In a phase II trial of Pivanex in patients with previously treated advanced NSCLC, only 3 out of 47 patients had partial responses (Reid et al., 2004). Twelve percent of patients experienced grade 3/4 toxicity including fatigue, dyspnea, and chest pain. Another phase II study of single agent romidepsin in previously treated advanced NSCLC did not show any objective responses despite transient stabilization of disease in some patients, enhanced acetylation of H4, and increased p21 expression (Schrump et al., 2008). Similarly, vorinostat monotherapy in patients with relapsed NSCLC failed to show any objective tumor responses and was associated with significant toxicity, including 28% grade 3/4 adverse events such as cytopenias and fatigue, and one possibly treatment related death (Traynor et al., 2009).

The mechanisms underlying the lack of clinically meaningful antitumor activity of HDACi remain speculative at this time, including a hypothesis that the resistance to HDACi is a critical evolutionary consequence of environmental exposure to HDACi and that only those cancer cells that have developed mutations that alter this response are inhibited by HDACi (Halsall and Turner, 2016). HDACi demonstrate synergy with not only conventional treatment modalities such as chemotherapy and radiation, but also molecularly targeted therapies, immune checkpoint inhibitors, and other epigenetic therapies. Consequently, most clinical trials have focused on combination strategies to harness the full therapeutic potential of HDAC inhibition in lung cancer.

### Combination Therapies Utilizing HDACi in NSCLC

#### Combination of HDACi With Cytotoxic Chemotherapy

Mounting evidence has demonstrated the synergistic activity of HDACi with cytotoxic chemotherapy. HDACi in combination with paclitaxel exerts synergistic anti-tumor effect via induction of p53 and tubulin hyperacetylation as well as prevention of upregulation of p21 (Zuco et al., 2011). Similar synergistic effect was observed with HDACi in combination with vinorelbine and platinum via increased expression of CHK2, CHK1, p21, and p27 leading to cell-cycle arrest and increased apoptosis (Gavrilov et al., 2014; Groh et al., 2015). Interestingly, paclitaxel resistant NSCLC cells demonstrate overexpression of HDAC1 and co-treatment with HDACi SNOH-3 and paclitaxel overcomes paclitaxel resistance (Wang et al., 2016). Based on this pre-clinical evidence, a phase II clinical trial evaluating the combination of vorinostat with carboplatin and paclitaxel as a first line therapy for advanced NSCLC was conducted (Ramalingam et al., 2010). In this randomized, double-blind, placebo-controlled trial, patients were randomized to receive conventional doses of carboplatin and paclitaxel with either vorinostat 400 mg daily or placebo, given on days 1 through 14 of each 21-day cycle for a maximum of 6 cycles. The response rate was higher in vorinostat arm compared to placebo (34 vs. 12%, *p* = 0.02). Median progression free survival (PFS) and overall survival (OS) were numerically superior in the vorinostat arm; however, the difference was not statistically significant. Addition of vorinostat was associated with higher toxicity including nausea, vomiting, fatigue, dehydration, and hyponatremia. Notably, 18% of patients on vorinostat developed grade 4 thrombocytopenia compared to 3% on the placebo arm (*p* ≤ 0.05). Another phase I trial evaluated combination of belinostat with carboplatin and paclitaxel. In this study, patients with chemotherapy-naïve advanced NSCLC received IV belinostat on days 1–5 of each 21-day cycle in combination with standard dose carboplatin and paclitaxel on day 3 of each cycle for up to 6 cycles. The most frequent adverse events were fatigue, nausea, diarrhea, and neutropenia. Median PFS was 5.7 months. The objective response rate was 35%, all responses being partial responses (Waqar et al., 2016). A phase I trial combining panobinostat with standard doses of carboplatin and etoposide was terminated because of prohibitive side effects of severe thrombocytopenia and febrile neutropenia at the lowest dose of panobinostat (Tarhini et al., 2013). These studies indicate that while the combination of chemotherapy with HDACi potentially offers a therapeutic advantage, the toxicity of these agents, especially myelosuppression and GI toxicity, prevent a wider application of the strategy in clinical practice. In order to leverage the synergistic therapeutic potential and to make side effect profile more favorable, future clinical trials should utilize more selective HDACi and explore sequential administration of these agents, where patients don’t receive simultaneous treatment with HDACi and cytotoxic chemotherapy. Preclinical studies have shown that the cells arrested at the G1/S checkpoint by cisplatin were more sensitive to subsequent treatment with HDAC inhibitors (Sato et al., 2006).

#### Combination of HDACi With Immune Checkpoint Inhibitors (ICI)

Immune checkpoint inhibition, either as single agent or in combination with cytotoxic chemotherapy, has become the standard of care first line treatment for advanced NSCLC (Gandhi et al., 2018; Paz-Ares et al., 2018; Reck et al., 2019). While a small subset of patients experiences remarkably durable disease responses, the responses in the remaining majority of the patients are short lived. One of the mechanisms of primary or acquired resistance to immune checkpoint inhibition is the paucity of T-cells in the tumor microenvironment and loss of tumor neoantigens (Herbst et al., 2014; Tumeh et al., 2014; Anagnostou et al., 2017). There is a growing interest in enhancing or restoring responses to ICI through epigenetic modulation of the tumor microenvironment (Beg and Gray, 2016; Weintraub, 2016). The interest in the combination of HDACi with ICI was initiated by a study that evaluated dual epigenetic modulation with entinostat and azacitidine. While the combination did not yield expected anti-tumor response, a subset of these patients subsequently went on to receive nivolumab. Five out of the six NSCLC patients showed a progression-free survival of 6 months post-treatment. This was a remarkable outcome for patients who had previously progressed on an ICI (Banik et al., 2019). HDACi have been shown to prime the tumor microenvironment for response to ICI through multiple mechanisms, including upregulation of MHC expression, T cell functionality, tumor antigens, T-cell chemokines, stimulatory effects on T cells, and the inhibition of suppressive cell types such as myeloid-derived suppressor cells (Vo et al., 2009; Kim et al., 2014; Zheng et al., 2016; Orillion et al., 2017; Topper et al., 2017). Analysis of azacitidine-induced pathways in The Cancer Genome Atlas (TCGA) project by mapping the derived gene signatures in NSCLC tumors has showed that azacitidine upregulates genes and pathways related to both innate and adaptive immunity and genes related to immune evasion (Wrangle et al., 2013). Additionally, dual HDAC and HSP90 inhibition decreases PD-L1 expression in IFN-gamma treated lung cancer cells suggesting its impact on modulating immunosuppressive ability of the tumor (Mehndiratta et al., 2020). A phase I/Ib study evaluated combination of vorinostat with PD-1 inhibitor pembrolizumab in patients with advanced NSCLC (Gray et al., 2019). Patients were either ICI-naïve or ICI-pretreated in the initial phase but had to be ICI-pretreated for phase Ib portion of the study. The treatment consisted of standard dose pembrolizumab 200 mg IV every 3 weeks plus vorinostat 200 or 400 mg per day. No dose limiting toxicities were observed. Fatigue and nausea/vomiting were the most common side effects (33 and 27%, respectively). Of the total 30 evaluable patients (6 ICI-naïve, 24 ICI-pretreated), 4 (13%) had partial response and 16 (53%) had stable disease, leading to a disease control rate of 67%. In the ICI-pretreated cohort, three patients had partial response and 10 had stable disease. The results of this early phase study are very encouraging for further evaluation of this combination in ICI pretreated patient population. The long-term outcomes of patients treated on this study and the results of multiple other ongoing studies evaluating combination of various other HDACi (entinostat, panobinostat, mocetinostat, abexinostat) with ICI are awaited.

#### Combination of HDACi With Tyrosine Kinase Inhibitors

Approximately 15% of advanced NSCLC tumors harbor sensitizing mutation in Epidermal Growth Factor Receptor (EGFR) and show marked response to EGFR tyrosine kinase inhibitors (TKIs). Despite the dramatic initial responses, most patients eventually develop resistance to the TKIs. One of the resistance mechanisms is decreased activity of Bcl2-like protein 11 (BIM). BIM is a proapoptotic molecule and its upregulation is essential for the induction of apoptosis in EGFR mutated lung cancer cells treated with an EGFR TKIs (Faber et al., 2011; Costa et al., 2014). A functional BIM deletion polymorphism is associated with inferior outcomes with EGFR-TKIs in EGFR mutated NSCLC (Ng et al., 2012; Isobe et al., 2014). Takeuchi et al. conducted a phase I trial of HDACi vorinostat in combination with gefitinib in BIM deletion polymorphism harboring EGFR-mutated NSCLC (Takeuchi et al., 2020). Twelve patients with advanced EGFR-mutated NSCLC, previously treated with an EGFR TKI and chemotherapy, were treated with gefitinib and escalating dose of vorinostat. The combination was well-tolerated and resulted in a 6 weeks disease control rate of 83.3%, which is notable since these patients previously had a disease progression on an EGFR TKI. Although median PFS was 5.2 months, median OS on this small early phase trial was encouraging at 22.1 months. Similarly, combination of HDACi panobinostat with third generation EGFR TKI osimertinib has been shown to enhance the induction of apoptosis and decrease the survival of osimertinib resistant cell lines and xenograft models, including those harboring C797S mutations, via elevation of BIM (Zang et al., 2020).

Another postulated mechanism of resistance to EGFR TKI is emergence of subpopulation of tumor cells with cancer stem cell like properties and HDAC sirtuin-1 (SIRT1) mediated survival advantage. Consequently, administration of a SIRT1 inhibitor tenovin6 (TV6) in combination with gefitinib showed tumor regression in resistant xenograft models. Additionally, co-administration of TV6 leads to decrease in the dose of gefitinib necessary to induce tumor response in preclinical models (Sun et al., 2020). A phase I/II trial enrolled 132 patients with advanced EGFR mutant NSCLC and randomized them to erlotinib plus entinostat or erlotinib plus placebo (Witta et al., 2012). Entinostat based combination led to superior OS in the subset of patients with high E-cadherin levels (9.4 vs. 5.4 months; *p* = 0.03), indicating potential role of E-cadherin as a biomarker for selecting patients for the treatment with erlotinib and entinostat.

#### Combination of HDACi With Radiation

Ionizing radiation exerts its anti-tumor effect through development of single–strand breaks, double-strand breaks (DSBs), and inter-strand crosslinks (Ward, 1988). DNA damage response pathways, specifically homologous recombination (HR) and non-homologous end joining (NHEJ), are activated in response to DSBs (Moynahan et al., 1999; Chapman et al., 2012). Upregulation of these pathways is implicated as one of the putative mechanisms for resistance to conventional ionizing radiation. HDACi upregulate γH2AX, an established marker of DSBs, in lung cancer cell lines in conjunction with ionizing radiation (Geng et al., 2006; Cuneo et al., 2007; Samuni et al., 2014). Additionally, HDACi downregulate the expression of RAD51, CHK1, and BRCA2—key DNA damage response pathway genes mediating repair of radiation-induced DNA damage (Brazelle et al., 2010; Huang et al., 2014). Additionally, HDAC inhibition leads to acetylation of Ku70/80 and XRCC4, rendering the NHEJ pathway defective (Miller et al., 2010). To build on the pre-clinical evidence, several clinical trials are underway utilizing combination of HDACi with ionizing radiation in NSCLC.

#### Combination of HDACi With Other Epigenetic Therapy

Combination therapy with HDACi with DNA methyltransferase inhibitors is based on robust preclinical data showing promotor hypermethylation as a key epigenetic even in lung cancer initiation and progression (Witta, 2012). Stage I NSCLC harboring hypermethylation of two of the four genes, *CDKN2a*, *CHD13*, *APC*, or *RASSF1a*, has been demonstrated to be associated with poor survival outcomes (Brock et al., 2008). In a phase I/II trial of 5-azacitidine and entinostat in heavily pre-treated advanced NSCLC, 1 out of 34 evaluable patients had a complete response that lasted for 14 months (Juergens et al., 2011). One patient had partial response, and 10 had stabilization of disease that lasted at least 12 weeks. Demethylation of the four genes, *CDKN2a, CDH13, APC*, and *RASSF1a*, detected in serial blood samples was associated with improved PFS (*p* = 0.034) and OS (*p* = 0.035) with the combination, indicating their potential role as predictive biomarkers for the benefit from treatment with HDACi and hypomethylating agents. Adjuvant treatment with 5-azacitidine and entinostat prolongs disease free survival (DFS) and OS in mice models following removal of primary lung, breast, and esophageal tumors, by inhibiting the trafficking of myeloid derived suppressor cells through downregulation of CCR2 and CXCR2 leading to disruption of premetastatic niches and inhibition of development of metastatic disease (Lu et al., 2020). Based on this finding, two trials evaluating the role of azacitidine and entinostat as adjuvant and neoadjuvant therapy for resectable NSCLC were initiated. However, these trials were terminated early because of slow accrual.

Table 1 summarizes notable completed and ongoing clinical trials utilizing HDACi in NSCLC.

**TABLE 1:** Select completed and ongoing clinical trials evaluating efficacy of HDACi in NSCLC.

Completed trials				

HDAC inhibitor	Regimen	Trial design	Efficacy	Toxicity
**Monotherapy**				
Pivanex (Reid et al., 2004)	Pivanex: 2.34 gm/m^2^/d IV on days 1,2,3 of a 21-day cycle	Phase II Previously treated advanced NSCLC (*n* = 47)	ORR 6.4% SD 30% mPFS 1.5 mo mOS 6.2 mo	Grade 3 and 4 toxicity in 6 patients each, including fatigue, asthenia, dyspnea, and chest pain
Romidepsin (Schrump et al., 2008)	Romidepsin: 17.8 mg/m^2^ IV on days 1 and 7 of a 21-day cycle	Phase II Previously treated advanced NSCLC (*n* = 19)	No objective responses. Ten patients had transient stabilization of disease.	Four patients had grade 3, 4 patients had grade 3/4 neutropenia, 1 patient had grade 4 thrombocytopenia.
Vorinostat (Traynor et al., 2009)	Vorinostat: 400 mg/day orally	Phase II Previously treated advanced NSCLC (*n* = 16)	No objective responses. mTTP 2.3 mo mOS 7.1 mo	One possible treatment related death, two grade 4 toxicities, 13 occurrences of grade 3 toxicities
Entinostat (Ryan et al., 2005)	Entinostat: Orally once a day or once every 14 days (q14-day) schedule with dose escalation	Phase I Previously treated advanced solid tumors (*n* = 31; 4 NSCLC patients)	No PR or CR. One NSCLC, 1 cervical cancer, and 2 melanoma patients had stable disease	Daily dosing intolerable. Q14-day schedule better tolerated. DLT—nausea, vomiting, anorexia, fatigue.
**Combination with chemotherapy**				
Vorinostat (Ramalingam et al., 2010)	Vorinostat 400 mg/day orally or placebo on days 1–14 + Chemotherapy (Carboplatin AUC 6 + Paclitaxel 200 mg/m^2^) IV on day 3 of each 21-day cycle, for maximum of 6 cycles	Randomized phase II Previously untreated advanced NSCLC (*n* = 94)	ORR: 34% with vorinostat vs. 12.5% with placebo (*p* = 0.02) mPFS: 6 months vs. 4.1 months (*p* = 0.48) mOS: 13 months vs. 9.7 months (*p* = 0.17)	Grade 4 thrombocytopenia, nausea, vomiting, fatigue, dehydration, and hyponatremia more frequent in vorinostat arm.
Belinostat (Waqar et al., 2016)	Belinostat IV on days 1–5 (starting at 1,000 mg/m^2^ dose) + Chemotherapy (Carboplatin AUC 6 + Paclitaxel 200 mg/m^2^) IV on day 3 of each 21-day cycle, for maximum of 6 cycles	Phase I Previously untreated advanced NSCLC (*n* = 23)	MTD 1,400 mg/m2 ORR: 35% mPFS: 5.7 mo	Most frequent adverse events: fatigue (91%), nausea (78%), constipation (74%) anemia, and diarrhea (65%), neutropenia (61%) dizziness, vomiting (57%), headache (52%)
Panobinostat (Tarhini et al., 2013)	Panobinostat orally 3 times a week (2-weeks on/1 week off) + Carboplatin AUC 5 on day 1 + Etoposide 100 mg/m^2^ IV on days 1–3 of each 21-day cycle for maximum of 6 cycles -> followed by panobinostat maintenance.	Phase I Previously treated advanced NSCLC (*n* = 6)	–	Two of the first 6 patients at the lowest dose level of panobinostat experienced DLT—grade 4 thrombocytopenia and grade 4 febrile neutropenia. Study was terminated.
**Combination with hypomethylating agent**				
Entinostat (Juergens et al., 2011)	Entinostat 7 mg/day orally on days 3–10 + Azacitidine SQ 30 mg/m^2^/d in 3 patients and 40 mg/m^2^/d in 42 patients on days 1–6 and 8–10 of each 28-day cycle	Phase I/II Previously treated advanced NSCLC (*n* = 45)	One patient had CR lasting for 14 months. One patient had PR lasting for 8 months. SD: 22% mOS among patients who received at least one cycle of therapy: 8.6 mo	No DLTs. Grade 3 or 4 toxicities in 28% of patients during cycle 1. Most common grade 3 or 4 toxicity was fatigue (12.5%).
**Combination with radiation**				
Vorinostat (Choi et al., 2017)	Vorinostat (200, 300, 400 mg/day) orally for 14 days + SRS for brain metastasis on day 3	Phase I NSCLC with up to 4 brain metastasis, ≤2 cm in size. (*n* = 17)	No local failures with median follow-up of 12 months	No DLT MTD: Vorinostat 400 mg/day Acute adverse events were reported by 10 patients (59%). Five patients discontinued vorinostat early and withdrew from the study. The most common reasons for withdrawal were dyspnea (*n* = 2), nausea (*n* = 1), and fatigue (*n* = 2).
	Vorinostat (200, 300, 400 mg/day) orally per RT fraction + Palliative thoracic radiation (30 Gy over 2 weeks)	Phase I (*n* = 17)	–	No DLT Most common non-serious adverse events: Anemia (12.5%) and fatigue (12.5%) (Results available on clinicaltrials.gov NCT00821951)
**Combination with ICI**				
Vorinostat (Gray et al., 2019)	Vorinostat (200 or 400 mg/day) orally + Pembrolizumab 200 mg IV every 3 weeks	Phase I/Ib ICI-naïve and -pretreated advanced NSCLC patients in phase I, ICI-naïve patients only in phase Ib (*n* = 33)	ORR: 13% SD: 53% ICI-pretreated patients: ORR-12.5%, SD-42%	No DLT RP2D dose: Pembrolizumab 200 mg IV every 3 weeks + Vorinostat 400 mg/day Any-grade adverse events were mainly fatigue (33%) and nausea/vomiting (27%).
**Combination with EGFR TKIs**				
Panobinostat (Gray et al., 2010)	Panobinostat + Erlotinib – with various dosing schedule	Phase I Previously treated NSCLC and head and neck cancer patients. EGFR alteration not required. (*n* = 15)	Of 12 evaluable patients, 7 had SD and 5 had PD.	The most common toxicities were rash (73%), nausea (67%), fatigue (67%), and diarrhea (47%). Grade 3/4 toxicities included nausea, neutropenia, and QTc prolongation. RP2D: Panobinostat 30 mg twice weekly for 2 weeks) and earlotinib 100 mg daily
Vorinostat (Takeuchi et al., 2020)	Vorinostat dose escalation (200, 300, 400 mg/day) on days 1–7 + Gefitinib 250 mg/day on days 1–14 of each 14-day cycle until disease progression	Phase I BIM deletion polymorphism/EGFR mutation double-positive NSCLC (*n* = 12)	mPFS: 5.2 mo 6-weeks DCR: 83.3%	No DLT RP2D of Vorinostat: 400 mg/day Treatment-related grade 3 adverse events included grade 3 hypokalemia (17%), lung infection and thrombocytopenia (8%) No treatment-related death or grade 4 adverse events were observed.
Entinostat (Witta et al., 2012)	Erlotinib 150 mg/day on days 1–28 + Entinostat 10 mg/day orally on days 1 and 15 of each 28-day cycle (EE) or Erlotinib + Placebo (EP)	Randomized phase II Previously treated patients with stage IIIB/IV non–small-cell lung cancer, no prior EGFR-TKIs (*n* = 132)	ORR: 3% with EE vs. 9.2% with EP (*p* = 0.13) mPFS: 1.97 months with EE vs. 1.88 with EP (*p* = 0.98) mOS: 8.9 months with EE vs. 6.7 months with EP (*p* = 0.39). In subgroup of patients with high E-cadherin, OS 9.4 months with EE vs. 5.4 months with EP (*p* = 0.03)	Rash, fatigue, diarrhea, and nausea the most common AEs in both groups. Percentage of patients with a serious AE (EE, 49.2% vs. EP, 46%) or with an AE leading to treatment discontinuation (EE, 43.1% vs. EP, 42.9%) were similar between groups.

**Ongoing trials**				

**HDAC inhibitor**	**Regimen**	**Trial design**	**Clinicaltrials.gov identifier**	

Vorinostat	Vorinostat + Pembrolizumab	II	NCT02638090	
Entinostat	Entinostat + Pembrolizumab	II	NCT02437136	
	Entinostat + Azacitidine + Nivolumab	II	NCT01928576	
Panobinostat	Panobinostat + Anti PD-1 antibody PDR001	I	NCT02890069	
Mocetinostat	Mocetinostat + Nivolumab	II	NCT02954991	
ACY-241 (Citarinostat)	ACY-241 + Nivolumab	I	NCT02635061	
Abexinostat	Abexinostat + Pembrolizumab	I	NCT03590054	

*ORR, Objective response rate; SD, Stable disease; PFS, Progression free survival; OS, Overall survival; TTP, Time to progression; PR, Partial response; CR, Complete response; DLT, Dose limiting toxicity; MTD, Maximum tolerated dose; RP2D, Recommended phase 2 dose; RT, Radiation therapy; ICI, Immune checkpoint inhibitor; TKI, Tyrosine kinase inhibitor.

## Challenges and Future Directions

Despite encouraging results from numerous preclinical and early clinical studies evaluating combination of HDACi with several other established or emerging treatment strategies, the utility of HDACi in the treatment of NSCLC remains exploratory. There are no randomized phase III trials utilizing HDACi in NSCLC. One of the major challenges is the toxicity profile of these agents, especially when combined with cytotoxic chemotherapy. HDACi are associated with several collateral toxicities on account of their widespread impact on a multitude of key cellular functions and limited selectivity for tumor cells. Of the four classes of HDACs, class 1 HDACs (HDAC 1, 2, 3, and 8) are primarily involved in promoting carcinogenesis and metastasis, and are the most well-studied HDACs, while class IV HDAC is the most poorly understood HDAC. Given the heterogeneity of various HDACs and their role in regulating genes involved in different cellular pathways, development of more selective HDACi, preferably HDAC class I inhibitors, with potent anti-tumor activity and more favorable side effect profile is desirable. Recent development of technologies to utilize nanocarriers, such as polymeric nanoparticles, PEG-coated nanoparticles, colloid carrier systems, PLGA nanoparticles, and albumin microspheres, are being investigated in clinical studies to deliver HDACi with enhanced solubility, tumor specificity and less toxicity (Enriquez et al., 2013; Martin et al., 2013; Goswami et al., 2018; Bertrand et al., 2019; Lee et al., 2019). Moreover, the optimum timing of administration of HDACi with other treatments remains unknown. The results of most of the early clinical trials are heterogeneous with only a subset of patients benefiting from HDACi based therapies.

A logical approach for future studies would be to develop strategies to mitigate some of the toxicities of HDACi by development of more tumor selective HDACi and explore different timing of administration of HDACi. Development of predictive biomarkers to allow better patient selection and consideration of variable impact of expression of different classes of HDACs on the prognosis of NSCLC will be of paramount importance. Additionally, the synergistic anti-tumor activity of HDACi with a number of anti-cancer therapies, such as chemotherapy, ICIs, radiation, and targeted therapies, suggests that combination strategies with multiple agents should be explored. The combinations of ICI with chemotherapy, EGFR TKIs with chemotherapy, and ICI with radiation have been shown to offer significant therapeutic advantage for NSCLC. Therefore, development of clinical trials incorporating selective HDACi with the already established combinations is a logical path forward. Finally, it is imperative to keep in mind the early pre-clinical evidence that in certain tumor types HDACi may in fact promote tumor cell migration and metastasis.

## Conclusion

HDAC driven epigenetic modulation is emerging as one of the key mechanisms promoting carcinogenesis and metastasis, making HDAC a potential target for cancer therapy. While HDACi are not highly efficacious as single agents for the treatment of NSCLC, the results of early phase clinical trials utilizing combination strategies have been encouraging, especially the combination with ICI and TKIs. Nonetheless, side effect profile of HDACi and their combination with chemotherapy is a challenge. Additionally, optimum timing of HDACi administration in the context of combination therapy is an area of ongoing research. Development of nanocarrier technologies for delivery of HDACi is an exciting step toward improving targeted delivery of these drugs. Finally, development of more selective HDACi and exploring the predictive biomarkers to guide patient selection for HDACi based therapy is imperative for continued future development of these agents. Ultimately, the answer to the question of whether HDAC inhibition is a hope or mere hype as a treatment strategy for NSCLC awaits results of multiple ongoing clinical trials.

## Author Contributions

HM and SJ contributed to the literature search and writing of the manuscript. Both authors contributed to the article and approved the submitted version.

## Conflict of Interest

The authors declare that the research was conducted in the absence of any commercial or financial relationships that could be construed as a potential conflict of interest.
