# Supplementary material for: Histone Deacetylase Inhibition in Non-small Cell Lung Cancer: Hype or Hope?
Source: Front Cell Dev Biol. 2020 Oct 9;8:582370. doi: 10.3389/fcell.2020.582370 (PMC7581936; doi:10.3389/fcell.2020.582370)
Supplement: Supplementary file 1 [file Data_Sheet_1.PDF]

Supplementary Table 1: HDAC targets of clinically relevant HDACi

HDAC Inhibitor	HDAC Target Class
Pivanex	Class I, II
Romidepsin	Class I
Vorinostat	Class I, II, IV
Entinostat	Class I
Belinostat	Class I, II, IV
Panobinostat	Class I, II, IV
Mocetinostat	Class I, IV
ACY-241 (Citarinostat)	Class II (selective HDAC6 inhibitor)
Abexinostat	Class I, II
